# 상급종합병원 병동 간호사의 환자안전 문화인식, 잡 크래프팅, 간호순회인식이 환자안전 관리활동에 미치는 영향

**DOI:** 10.7586/jkbns.24.020

**Published:** 2024-08-26

**Authors:** Saet-Byeol Kim, Yun-Hee Kim

**Affiliations:** Department of Nursing, Pukyong National University, Busan, Korea; 부경대학교 간호학과

**Keywords:** Patient safety, Culture, Safety management, Perception, Nursing, 환자안전, 문화, 안전관리, 인식, 간호

## Abstract

**Purpose:**

This study investigated patient safety management activities among general hospital nurses and aimed to identify the factors influencing patient safety management activities.

**Methods:**

A cross-sectional descriptive design included 159 ward nurses working at two tertiary hospitals located in Busan city. Data were collected with self-reported questionnaires from April 8 to April 20, 2024 and were analyzed using descriptive statistics, the t-test, analysis of variance, Pearson correlation coefficients, and multiple regression in SPSS 29.0.

**Results:**

Factors significantly influencing patient safety management activities in nurses were perceptions of patient safety culture (β = .47, *p* < .001) and perceptions of patient rounding (β = .31, *p* < .001). The explanatory power of these variables was 43%.

**Conclusion:**

This study found that perceptions of patient safety culture and perceptions of patient rounding had significant effects on nurses’ patient safety management activities. Thus, it is necessary to promote patient safety management activities among nurses through the development of intervention programs to improve perceptions of patient safety culture and perceptions of patient rounding.

## 서론

### 1. 연구의 필요성

환자안전은 간호의 민감성 질 지표로 간호의 가치를 평가하고 간호의 우수성을 나타내는 지표로 중요하며[1] 낙상, 욕창, 병원 내 감염은 간호의 질을 평가하는데 자주 사용되는 지표이다[2]. 국내에서는 환자안전과 의료의 질 향상을 위해 2011년부터 의료기관 인증제를 도입하여 의료기관이라면 마땅히 환자안전 보장을 위해 노력해야 한다는 기본 전제하에 최근 2021년 4주기 인증평가를 시행하였으며, 환자안전 관리활동의 중요성이 점차 강조되고 있다[3].

환자안전 사고 예방을 위한 이러한 국가적 차원의 노력에도 불구하고 2018년부터 2022년까지 5년간 환자안전 사고는 총 63,088건으로 지속적으로 증가하였으며 최근 2022년에는 14,820건으로 가장 많이 보고되었다[4]. 또한 의료정보기술의 발달로 의료서비스 접근 기회가 점차 증가하고 있으나 수명연장으로 인해 입원 환자의 중증도 및 복잡성 증가하고 있어 예기치 못한 안전사고가 발생하고 있다[5]. 이러한 환자안전 사고는 매우 복잡하고 범위가 넓으며[6], 환자안전 사고의 증가는 병원의 재정적 손실, 재원 기간의 연장, 의료진과 병원에 대한 신뢰감 저하, 환자의 생명과 건강에 치명적인 영향을 줄 수 있다[7]. 이에 환자안전 사고를 예방하고 환자안전의 질을 높이기 위해 의료기관들의 자발적인 환자안전관리활동은 중요하다고 할 수 있다[8].

환자안전 관리활동은 병원환경을 보다 안전하게 개선하고 사고를 예방하는 활동으로서 환자안전을 위한 병원 내의 모든 활동이다[9]. 간호사의 환자안전 관리활동은 의료의 질과 환자안전에 긍정적인 영향을 미치는 것으로 나타났다[10]. 간호사는 24시간 현장에서 환자를 직접 돌보며 환자안전과 가장 밀접한 관련이 있으므로[11], 환자안전에 관심을 가지고 환자안전 사고를 최소화하기 위해 중요한 역할을 해야 하는 전문직이다[12]. 병원에서 발생하는 환자안전 사고 중 투약, 감염, 낙상 등의 간호활동에서의 사고가 가장 큰 비중을 차지하고 있으며 전체 안전사고의 약 50%에 이르는 것으로 나타났다[4]. 간호사는 다수의 환자를 돌보며 안전사고 위험 사정과 중재 수행을 통해 환자안전 향상에 크게 기여하고 있으므로[13], 간호사의 환자안전 관리활동을 파악하는 것은 중요하다. 국내 간호사의 환자안전 관리활동의 영향요인으로 알려진 변수는 환자안전 문화인식, 간호근무환경, 직무몰입, 직무만족, 직무스트레스 등이 있으며[12,14,15], 환자안전 관리활동 향상을 위해 환자안전 관리활동에 영향을 주는 요인들을 지속적으로 파악할 필요가 있다.

환자안전 문화인식은 환자중심의 의료와 간호를 가능하게 하는 환자안전의 핵심 요소로[16], 의료전달과정에서 발생할 수 있는 의료 오류를 예방하고 환자에게 가능한 위해사건이 발생되지 않도록 하기 위한 조직, 부서, 개인 차원에서 공유하고 있는 신념, 가치, 행동 패턴을 말한다[17]. 국내외 여러 나라에서 환자안전 문화인식을 환자안전 향상을 위한 전략으로 환자안전 문화를 측정하고 개선하기 위해 노력하고 있다[18,19]. 긍정적인 환자문화인식은 환자안전 관리활동 수준을 향상시키는 주요 변수로 알려져 있으며[9,12], 환자안전 문화인식 수준이 높을수록 환자안전사고를 감소시켰다[20]. 환자안전 문화인식은 조직문화의 일부로 의료기술의 변화와 세대 교체와 같은 시간의 흐름에 영향을 받을 수 있으므로 환자안전 관리활동의 중요한 영향요인으로서 지속적으로 파악할 필요가 있다.

잡 크래프팅은 간호사의 직무몰입을 높여 환자안전을 향상시켰으며[21], 잡 크래프팅 활동을 많이 할수록 간호사의 환자안전 관리활동을 향상시키는 것으로 나타났다[22]. 잡 크래프팅은 개인이 자신의 역량에 맞게 자기 주도적으로 직무를 재구성하는 일련의 활동을 말하며, 흥미와 관심을 가지고 직무요구와 자원의 균형을 만들어가기 위해 직무나 관계영역을 스스로 변화시켜 나가는 신체적, 인지적 행동이다[23]. 간호사의 잡 크래프팅은 직무만족과 조직몰입을 높이며[24,25], 조직몰입과 직무만족이 높은 간호사는 환자안전 관리활동을 잘 수행하는 것으로 나타났다[14,26]. 잡 크래프팅은 간호사의 직무만족, 재직의도 등과 관련되어 다양한 연구들이 진행되었으나 환자안전 관리활동과의 직접적인 관계를 파악한 연구는 부족한 실정이다.

간호순회는 환자를 정기적으로 확인하며 환자안전 위험을 예방할 수 있는 환자 치료에 대한 적극적이고 구조화된 활동으로[27], 간호순회인식은 간호순회에 대한 간호사의 가치, 신념 및 태도를 말한다. 간호순회에 대한 인식이 긍적적일수록 간호순회를 더 많이 하는 것으로 나타났다[28]. 간호순회가 많아질수록 낙상, 욕창, 카테터 관련 요로감염이 감소하는 것으로 나타났다[29]. 이러한 긍정적인 환자 결과에도 불구하고 간호순회는 불필요한 활동이라 인식되며 부족한 인력과 과다한 업무량 같은 체계적인 문제로 이행이 어렵다고 하였다[30]. 이에 간호순회인식과 환자안전 사이의 깊은 관련성을 바탕으로 간호순회인식 수준을 파악하고, 간호순회인식이 환자안전 관리활동의 직접적인 영향요인으로 작용하는지 살펴볼 필요가 있다.

앞서 선행연구에서 살펴본 바와 같이 잡 크래프팅과 간호순회인식은 환자안전 관리활동에 유의한 영향을 미칠 것으로 기대되나 환자안전 문화인식과 함께 그 영향을 다각도로 살펴본 연구는 찾기 어려웠다. 이에 본 연구는 상급종합병원 병동 간호사 대상으로 환자안전 문화인식, 잡 크래프팅 및 간호순회인식이 환자안전 관리활동에 미치는 영향을 규명하고, 상급종합병원 병동 간호사의 환자안전 관리활동을 향상시킬 수 있는 기초자료를 제시하여 임상간호학에서 요구되는 안전간호와 관련된 기초간호학적 지식을 수립하기 위하여 시도되었다.

### 2. 연구 목적

본 연구의 목적은 상급종합병원 병동 간호사의 환자안전 문화인식, 잡 크래프팅, 간호순회인식이 환자안전 관리활동에 미치는 영향을 파악하기 위함이며, 구체적인 목적은 다음과 같다.

첫째, 대상자의 환자안전 문화인식, 잡 크래프팅, 간호순회인식, 환자안전 관리활동의 정도를 파악한다.

둘째, 대상자의 일반적 특성에 따른 환자안전 관리활동의 차이를 파악한다.

셋째, 대상자의 환자안전 문화인식, 잡크래프팅, 간호순회인식과 환자안전 관리활동 간의 상관관계를 파악한다.

넷째, 대상자의 환자안전 관리활동에 영향을 미치는 요인을 파악한다.

## 연구 방법

### 1. 연구 설계

본 연구는 상급종합병원 병동 간호사의 환자안전 문화인식, 잡 크래프팅, 간호순회인식이 환자안전 관리활동에 미치는 영향을 확인하기 위한 서술적 조사연구이다.

### 2. 연구 대상

본 연구대상은 부산광역시에 소재하는 2개 상급종합병원 병동에 근무하는 간호사 중 임상근무경력이 6개월 이상인 자로, 본 연구의 목적을 이해하고 자발적으로 연구 참여에 동의한 자이다. 특수 파트에서 근무하거나 환자에게 직접 간호를 수행하지 않는 간호사는 제외하였다. 표본 수는 G-power 3.1.9.7 program을 이용하였으며, 다중회귀분석에서 유의수준 .05, 검정력 .90, 중간 정도 효과크기 .15, 예측변인 10개로 산출한 결과, 최소 표본크기는 147명이었으며 탈락률 20%를 고려하여 180명에게 설문지를 배부하였다. 그중 응답이 불충분한 설문지 21부를 제외하여 159부를 최종 분석에 사용하였다.

### 3. 연구 도구

#### 1) 대상자의 특성

대상자의 특성은 성별, 연령, 결혼상태, 교육수준, 총임상경력, 현부서 임상경력, 진료부서를 포함하였다.

#### 2) 환자안전 관리활동

환자안전 관리활동은 환자안전 실무표준을 기초로 하여 Lee [31]가 개발한 간호사의 환자안전관리활동을 측정하는 총 46문항의 도구에서 Lee [32]가 일부 문항을 수정·보완한 도구를 사용하였다. 총 40문항으로 문항은 환자확인 7문항, 투약간호 7문항, 구두처방 3문항, 수술·시술 4문항, 감염예방 3문항, 안전한 환경 3문항, 응급상황 7문항, 낙상예방 3문항, 욕창예방 3문항으로 구성되어 있다. 5점 Likert 척도로 ‘전혀 그렇지 않다’ 1점부터 ‘매우 그렇다’ 5점의 점수이며, 점수가 높을수록 환자안전 관리활동이 높음을 의미한다. Lee [31]의 연구에서 Cronbach`s α는 .92이었으며, Lee [32]의 연구에서 Cronbach`s α는 .92였다. 본 연구에서 Cronbach`s α는 .93이었으며 하부요인별 신뢰도 Cronbach`s α는 투약간호 .67에서 욕창예방 .91으로 나타났다.

#### 3) 환자안전 문화인식

환자안전 문화인식은 Lee [33]가 개발한 한국형 환자안전 문화 측정도구를 사용하였다. 이 도구는 총 35개 문항으로 환자안전 개선시스템 관련 4문항, 환자안전 정책 및 절차 관련 4문항, 환자안전 지식·태도 관련 5문항, 리더십 관련 9문항, 비처벌적 환경 관련 4문항, 팀워크 관련 6문항, 환자안전 우선순위 관련 3문항으로 구성되어 있다. 5점 Likert 척도로 각 문항은 ‘전혀 그렇지 않다’ 1점에서 ‘매우 그렇다’ 5점의 점수이며, 점수가 높을수록 환자안전 문화인식이 긍정적임을 의미한다. Lee [33]가 도구 개발 당시 신뢰도 Cronbach`s α는 .93이었고 본 연구에서 Cronbach`s α는 .93였다. Lee [33]의 연구에서 하부요인별 신뢰도 Cronbach`s α는 환자안전 우선순위 .66에서 리더십 .91까지 나타났다. 본 연구에서 하부요인별 신뢰도 Cronbach`s α는 환자안전 우선순위 .65에서 리더십 .90으로 나타났다.

#### 4) 잡 크래프팅

잡 크래프팅은 Slemp and Vella-Brodrick [34]이 기업 직장인을 대상으로 개발한 도구 Job Crafting Questionnaire (JCQ)를 Im 등[35]이 한국어로 번안한 한국판 잡 크래프팅 소명척도 (JCQ-K)를 사용하였다. 도구는 총 15개 문항으로 인지 크래프팅 5문항, 업무 크래프팅 5문항, 관계 크래프팅 5문항으로 구성되어 있다. 6점 Likert 척도로 ‘전혀 그렇지 않다’ 1점에서 ‘매우 그렇다’ 6점의 점수이며, 점수가 높을수록 잡 크래프팅의 정도가 높음을 의미한다. Slemp and Vella-Brodrick [34]가 도구 개발 당시 신뢰도 Cronbach`s α = .91, Im 등[35]의 연구에서 Cronbach`s α는 .93였다. 본 연구에서 Cronbach`s α는 .86였다.

#### 5) 간호순회인식

간호순회인식은 간호사의 간호순회인식를 측정하기 위해 Neville 등[36]이 개발한 Nurses` Perceptions of Patient Rounding Scale을 Han과 Kim [37]이 한국어로 번안한 도구를 사용하였다. 도구는 총 40문항으로 간호사의 이익 8문항, 의사소통 15문항, 환자의 이익 9문항의 3개 하부요인과 간호순회 일정 및 문화적, 윤리적 문제와 기밀유지 사항에 대한 8문항으로 구성되어 있다. 5점 Likert 척도로 ‘전혀 그렇지 않다’ 1점부터 ‘매우 그렇다’ 5점의 점수로, 점수가 높을수록 간호순회 인식 수준이 높음을 의미한다. Neville 등[36]이 도구를 개발한 당시 Cronbach`s α = .92였으며, Han과 Kim [37]의 연구에서 Cronbach`s α는 .86였다. 본 연구에서 Cronbach`s α는 .84였다.

### 4. 자료 수집

본 연구의 자료 수집은 2024년 4월 8일부터 4월 20일까지였으며, 부산시 소재 상급종합병원 2개소를 직접 방문하여 간호부에 연구목적과 자료수집을 설명하고 협조를 구하여 연구 참여를 희망하는 대상자에게 해당 부서장을 통하여 설문지를 배부하였다. 연구대상자에게는 연구 설명문을 통하여 본 연구의 목적과 절차, 개인정보의 비밀보장, 관련 자료는 익명으로 처리되며 연구결과는 연구 이외의 목적으로 사용되지 않음을 설명하고 연구 참여 도중 언제든지 중단할 수 있으며 이에 따른 불이익이 발생하지 않음에 대한 정보를 제공하였다. 자발적으로 참여하는 대상자에게 연구 참여동의서를 서면으로 받은 후 설문조사를 실시하였다. 설문에 소요되는 시간은 15분 정도였으며, 응답한 설문지는 밀봉된 봉투에 넣어서 해당 부서장에게 협조를 구하여 회수하였다. 연구에 참여한 대상자에게 소정의 답례품을 제공하였다.

### 5. 자료 분석

수집된 연구의 자료는 IBM SPSS/WIN 29.0 (IBM Corp., Armonk, NY, USA) 프로그램을 이용하여 분석하였다. 일반적 특성은 실수, 백분율, 평균, 표준편차로 분석하였으며, 환자안전 문화인식, 잡 크래프팅, 간호순회인식 및 환자안전 관리활동 정도는 평균과 표준편차로 분석하였다. 일반적 특성에 따른 환자안전 관리활동 차이는 Independent t-test와 ANOVA로 분석하였다. 대상자의 환자안전 문화인식, 잡 크래프팅, 간호순회인식과 환자안전 관리활동 간의 상관관계는 Pearson correlation coefficients로 분석하였으며, 환자안전 관리활동에 영향을 미치는 요인은 환자안전 관리활동과의 상관관계를 보인 환자안전 문화인식, 잡 크래프팅, 간호순회인식을 독립변수로 투입하여 다중회귀분석을 실시하였다.

### 6. 윤리적 고려

본 연구는 부경대학교 기관생명윤리위원회 승인(No. 1041386-202402-HR-20-02)을 받은 후 설문조사를 진행하였다. 연구에 참여하는 모든 대상자에게 연구의 목적과 절차, 연구 기간 등의 정보를 연구 설명문을 통해 설문 전에 안내하였으며 참여자가 원치 않을 때는 언제든지 참여 철회가 가능하고 이에 따른 불이익이 없음을 공지하였다. 또한 수집된 모든 정보는 연구 이외의 목적으로 사용되지 않으며 비밀이 보장될 것을 약속하였고 설문지를 시행하기 전 기재된 연구 설명문을 읽은 후 설문지를 작성하여 제출하면 연구 참여에 동의한 것으로 간주하였다. 수집된 자료는 연구목적으로만 사용되며 연구책임자만 접근가능하도록 비밀번호가 설정된 USB에 보관하였다. 연구와 관련된 모든 사항들은 보안과 익명성이 유지되고, 연구 종료 후 자료는 3년간 보관되며 이후 파일은 영구삭제로 모두 폐기할 예정이다.

## 연구 결과

### 1. 대상자의 일반적 특성과 환자안전 관리활동

본 연구대상자의 성별은 여성 155명(97.5%)이었으며 평균 연령은 29.35 ± 5.83세로 30세 미만이 104명(65.4%)이었다. 결혼 상태는 미혼이 124명(78.0%), 교육 수준은 4년제 대학 졸업이 138명(86.8%)으로 가장 많았다. 총임상경력은 평균 82.41 ± 73.36개월이었으며, 현부서 임상경력은 평균 44.29 ± 38.06개월이었다. 진료부서는 내과가 84명(52.8%)으로 가장 많았다.

대상자의 특성에 따른 환자안전 관리활동의 차이를 살펴보면 성별, 연령, 결혼 상태, 교육 수준, 총임상경력, 현부서 임상경력, 진료부서 모두 유의한 차이가 없었다(Table 1).

### 2. 환자안전 문화인식, 잡 크래프팅, 간호순회인식, 환자안전 관리활동 정도

대상자의 환자안전 문화인식의 정도는 평균 3.88 ± 0.43점이었다. 환자안전 문화인식의 하위영역 중 평균이 가장 높은 영역은 환자안전 지식 및 태도(4.25 ± 0.47점), 두 번째로 높은 영역은 리더십(4.09 ± 0.50점)이었고, 그 다음으로 팀워크(4.04 ± 0.51점), 환자안전 정책 및 절차(3.91 ± 0.60점), 환자안전 개선시스템(3.70 ± 0.52점) 순으로 나타났다. 반면에 가장 낮은 하위영역은 환자안전 우선순위(3.15 ± 0.77점)로 나타났다.

대상자의 잡 크래프팅 정도는 평균 3.93 ± 0.64점이었다. 가장 높은 하위영역은 인지 크래프팅(4.04 ± 0.85점)이었고, 관계 크래프팅(3.93 ± 0.77점), 업무 크래프팅(3.90 ± 0.78점) 순으로 나타났다.

대상자의 간호순회인식 정도는 평균 3.60 ± 0.30점이었다. 하위영역 중 환자의 이익(3.76 ± 0.33점)으로 가장 높았고 다음으로 의사소통(3.57 ± 0.47점), 간호사의 이익(3.50 ± 0.34점) 순이었다.

대상자의 환자안전 관리활동 정도는 평균 4.25 ± 0.41이었다. 가장 높은 하위영역은 낙상예방(4.59 ± 0.52점)이었으며, 욕창예방(4.58 ± 0.54점), 감염예방(4.49 ± 0.51점), 정확한 수술·시술확인(4.30 ± 0.57점), 정확한 구두처방(4.28 ± 0.63점), 안전한 환경(4.27 ± 0.59점), 정확한 환자확인(4.25 ± 0.43점), 응급상황관리(4.22 ± 0.58점) 순으로 높았다. 반면에 가장 낮은 하위영역은 투약간호(3.93 ± 0.50점)로 나타났다(Table 2).

### 3. 환자안전 문화인식, 잡크래프팅, 간호순회인식, 환자안전 관리활동의 상관관계

대상자의 환자안전 관리활동은 환자안전 문화인식(r = .60, *p* < .001), 잡 크래프팅(r = .16, *p* = .043), 간호순회인식(r = .54, *p* < .001)과 유의한 양의 상관관계가 있는 것으로 나타났다(Table 3).

### 4. 대상자의 환자안전 관리활동 영향요인

대상자의 환자안전 관리활동에 영향을 미치는 요인을 파악하기 위하여 다중회귀분석을 실시하였다. 본 연구결과 환자안전 관리활동과 유의한 상관관계를 보인 환자안전 문화인식, 잡 크래프팅, 간호순회인식을 분석에 투입하였다.

회귀분석 모형의 독립성 검증결과 Durbin-Watson 지수는 1.98로 2에 가까워 자기상관성의 문제가 없는 것으로 확인되었다. 잔차의 정규성과 등분산성을 검정하기 위해 정규확률 그래프와 잔차의 산포도를 확인한 결과 잔차가 45도 직선에 근접하며 잔차의 산점도는 잔차들이 0을 중심으로 고르게 분포하여 잔차의 성규성과 등분산성을 만족하였다. 각 변수들의 공차한계(tolerance)는 .64-.83으로 0.1 이상이었으며, 독립변수 간의 분산팽창인자(variance inflation factor)는 1.19-1.54로 10미만으로 다중공선성의 문제가 없는 것으로 확인되었다. 회귀분석 결과 간호사의 환자안전 관리활동 영향요인은 환자안전 문화인식(β = .47, *p* < .001), 간호순회인식(β = .31, *p* < .001)으로 나타났으며 변인의 설명력은 43%였다(F = 39.71, *p* < .001) (Table 4).

## 논의

본 연구는 상급종합병원 병동 간호사의 환자안전 관리활동에 영향을 미치는 요인을 파악하기 유위해 시행되었으며 다음과 같이 논의하고자 한다.

본 연구대상자의 환자안전 관리활동 정도는 4.25점(범위: 1-5점)으로 나타났다. 상급종합병원 간호사를 대상으로 같은 도구를 사용한 연구[38]에서도 4.13점으로 나와 본 연구와 유사하였다. 환자안전 관리활동의 하위영역 중에서 낙상예방 점수가 가장 높게 나타났는데 이러한 결과는 종합병원 간호사를 대상으로 한 연구[39]와 일치하였다. 낙상은 의료기관에서 흔하게 발생하는 환자안전 사고로[4], 환자안전 사고 유형 중 낙상사고 환자의 재원일수가 가장 크게 증가하는 것으로 나타났다[7]. 이에 현장에서 일하는 간호사는 낙상예방을 위한 교육을 필수로 받게 되며 이러한 교육이 낙상예방활동에 영향을 미친 것으로 파악된다[15]. 반면에 환자안전 관리활동에서 가장 낮은 점수를 보인 영역은 투약간호로 나타났는데, 상급종합병원 간호사를 대상으로 한 연구[38]와 일치하였다. 2022년 의료기관에서 발생했던 환자안전 사고를 살펴보면, 하위영역 중 투약이 43.3%로 가장 높았으며[4], 투약오류의 영향요인을 조사한 선행연구[40]에서 간호사의 직무 스트레스, 피로가 투약오류에 영향을 미치는 것으로 나타났다. 간호사는 교대근무 형태가 많고 전문적인 간호를 수행하며 다양한 직종과 상호작용을 하는 직무 특성으로 많은 피로와 스트레스를 경험하며[41], 간호사의 피로 정도가 증가하면 주의력이 감소했으며 피로는 투약오류의 영향요인으로 나타났다[42]. 또한 중증도와 복잡성이 높은 환자 간호라는 상급종합병원의 근무환경 특성은 직무스트레스에 많은 영향을 끼칠 것이라 사료된다. 따라서 투약간호와 관련된 환자안전 관리활동을 향상시키기 위해 간호사의 직무 스트레스와 피로를 개선할 수 있는 실질적인 방안이 필요할 것으로 생각된다.

본 연구 결과 상급종합병원 병동 간호사의 환자안전 관리활동에 영향을 미치는 요인은 환자안전 문화인식, 간호순회인식이었으며 환자안전 문화인식은 가장 큰 영향요인으로 확인되었다. 중소병원 간호사를 대상으로 한 연구[43]에서도 환자안전 문화인식은 환자안전 관리활동의 영향요인으로 나타나 본 연구를 뒷받침하였으며 환자안전 문화인식이 높을수록 환자안전 관리활동 정도가 높게 나타났다. 일반간호사를 대상으로 한 연구[15]에서 간호사의 환자안전 문화인식이 높을수록 환자안전 관리활동 중 낙상예방활동 수준이 높게 나타나 본 연구결과와 같은 맥락에서 해석될 수 있다. 본 연구 대상자의 환자안전 문화인식은 3.88점(범위: 1-5점)이었으며, 종합병원 간호사를 대상으로 같은 도구를 사용한 연구[44]에서도 3.81점으로 나와 본 연구와 유사하였다. 본 연구에서 환자안전 문화인식의 하위영역 중 환자안전 지식·태도의 점수가 가장 높게 나타났는데, 종합병원 간호사를 대상으로 한 연구[22]에서는 환자안전 지식·태도의 점수뿐만 아니라 환자안전 관리활동에 가장 많은 영향을 미치는 것으로 나타났다. 반면에 환자안전 우선순위 점수가 가장 낮게 나타났는데 환자안전 우선순위는 업무가 많거나 바쁜 경우에도 환자안전에 대한 원칙과 절차를 준수하는 것을 의미한다. 환자안전 우선순위가 가장 낮게 나타난 이러한 결과는 환자안전의 중요성을 잘 알고 있음에도 간호 인력부족으로 인해 실제 업무에서 환자안전 우선순위에 따른 안전절차를 지키지 못하는 것으로 보여진다[15,45]. 또한 환자안전 문화인식은 직무 스트레스와 환자안전 간호활동 간에 매개역할을 하는 것으로 나타났는데, 업무량 과중과 부적절한 대우와 보상에 대한 직무 스트레스가 과도하게 증가하면 환자안전 문화에 대해 부정적인 인식을 증가시킬 수 있을 뿐 아니라 환자안전 간호활동에 부정적인 효과를 나타내는 것으로 나타났다[12,46]. 따라서 간호사에 대한 적정한 업무량 배분과 적정 인력 수준의 배치, 성과에 따른 적절한 대우와 보상을 통해 직무 스트레스를 감소시키고, 환자안전 지식 및 태도에 대한 지속적인 교육을 통해 환자안전 문화인식과 환자안전 관리활동 수준을 향상시킬 수 있도록 조직의 관심과 지원정책이 필요하다.

간호순회인식은 환자안전 관리활동과의 관계를 분석한 연구가 부족하여 선행연구와의 직접적인 비교는 어려우나, 본 연구결과 간호순회인식은 상급종합병원 병동 간호사의 환자안전 관리활동에 영향을 미치는 요인으로 나타났다. 간호순회는 환자를 정기적으로 확인하며 낙상과 같은 환자안전 위험을 예방할 수 있는 환자 치료에 적극적이고 구조화된 접근 방식으로[27], 환자의 안전을 향상시키고 낙상, 욕창의 발생과 같은 부상을 줄이기 위해 취할 수 있는 환자안전 관리활동의 일환으로 보고되고 있다[29]. 이에 간호순회인식이 높을수록 간호순회 이행 정도가 높게 나타난 연구결과를 바탕으로[28,36], 간호순회인식과 환자안전 관리활동의 관련성을 확인할 수 있었다. 간호순회는 환자안전 및 만족도 향상 등의 환자 이익뿐만 아니라 환자로부터 콜벨 감소 등을 통해 간호 시간이 보장되고 환자 요구를 잘 파악할 수 있는 간호사의 능력 및 직원들 간의 의사소통 및 팀워크를 향상시켰다[30]. 본 연구 대상자의 간호순회인식은 3.61점(범위: 1-5점)이었으며 상급종합병원 간호사 대상으로 같은 도구를 사용한 연구[28]에서도 3.46점으로 나와 본 연구와 유사한 결과를 보였으며 본 연구에서 간호순회인식의 하위영역 중에서 간호사 이익이 가장 낮게 나타나 상급종합병원 간호사 대상으로 한 연구[47]를 뒷받침하였다. 간호순회인식은 업무량, 근무부서, 근무 인력의 영향을 받으며[30], 중증도가 높은 환자 간호로 간호순회 이행 어려움을 느끼는 것으로 나타났는데[28] 이는 국내 상급종합병원의 높은 업무량이 반영된 것으로 보인다[48]. 이에 환자안전 관리활동 수준을 높이기 위한 전략으로 간호순회가 잘 정착되려면 환자 중증도를 반영한 업무 조정 및 보조인력 지원 등 의료기관과 국가적 차원의 노력이 필요하다. 또한 환자와 간호사 모두에게 이익이 된다는 것을 간호사들에게 교육함으로써 간호사들이 간호순회를 간호업무에 통합하려는 의향을 자발적으로 갖도록 하는 것이 필요하다[28,49]. 이러한 중재를 고안하여 적용한다면 간호순회인식을 제고함으로써 환자안전 관리활동을 증진시킬 수 있을 것이라 생각된다.

본 연구결과 잡 크래프팅은 상급종합병원 병동 간호사의 환자안전 관리활동의 영향요인으로 유의하지 않아 종합병원 간호사를 대상으로 한 연구[22]와 다른 결과를 보여주었다. 종합병원 간호사 대상으로 한 연구[22]에서는 잡 크래프팅 정도가 높을수록 환자안전 관리활동을 많이 하는 것으로 나타났다. 이와 같은 맥락에서 간호사를 포함한 의료종사자들의 안전이행에 대한 연구[50]에서는 인지 크래프팅이 안전이행에 영향요인으로 나타났으며, 간호사들이 전문직으로서 자부심을 가지고 의사의 지시에 따른 수행만 한다고 생각하지 않고 환자를 살리고 더 나아가 타인의 삶에 도움이 될 수 있다는 생각을 통하여 업무에 대한 경계를 허무는 인지 크래프팅 활동이 안전이행에 중요한 영향요소라고 하였다. 본 연구 대상자의 잡 크래프팅은 3.96점(범위: 1-6)이었으며, 상급종합병원과 종합병원 간호사 대상으로 같은 도구를 사용한 연구[25]에서도 3.83점으로 나와 본 연구와 유사하였다. 반면에 종합병원 의료종사자를 대상으로 같은 도구를 사용한 연구[50]에서 간호사의 잡 크래프팅은 3.29점으로 본 연구보다 낮아 병원급별 점수 차이를 보였다. 본 연구에서는 상급종합병원 간호사만을 대상으로 하고 있어 추후 병원 규모별 잡 크래프팅 정도의 차이가 있는지 파악할 필요가 있다. 본 연구에서 잡 크래프팅과 환자안전 관리활동은 양의 상관관계로 나타났으며 환자안전 문화인식, 간호순회인식과는 더욱 높은 상관계수를 보였다. Empirical guidelines에 따르면 상관계수가 .30을 초과할 경우 높은 상관관계라 하였으며, 이에 잡 크래프팅이 환자안전 문화인식, 간호순회인식을 매개하여 환자안전 관리활동에 영향을 미치는지 추후 확인할 필요가 있다. 또한 본 연구에서 잡 크래프팅을 측정하기 위해 사용한 도구가 간호사를 대상으로 한 도구가 아닌 기업 직장인을 대상으로 개발된 도구[26]를 사용하여 실제 간호사의 잡 크래프팅과 차이가 있을 수 있다. 따라서 추후 연구에서는 간호사에게 적합한 도구를 사용하여 보다 정확한 측정이 요구된다.

본 연구는 상급종합병원 병동 간호사를 대상으로 환자안전 관리활동 정도를 파악하고, 이에 영향을 미치는 요인을 다각적으로 파악했다는데 의의가 있다. 또한 본 연구는 환자안전 문화인식, 간호순회인식이 상급종합병원 병동 간호사의 환자안전 관리활동의 영향요인으로 나타나 본 연구 결과를 토대로 환자안전 관리활동을 증진시키기 위한 기초자료로 활용되어 임상에서 안전간호와 관련된 기초학적 지식 수립 및 환자안전 관리활동 중재 프로그램 개발 도움이 될 수 있을 것이라 본다. 하지만 본 연구는 일부 지역에 국한된 상급종합병원 병동 간호사를 대상으로 하는 서술적 조사 연구로 일반화함에 있어 신중을 기해야 할 것이다. 따라서 추후 연구에서는 지역 및 대상 병원을 확대하여 비교, 반복 연구할 필요가 있다.

## 결론

본 연구에서 상급종합병원 병동 간호사의 환자안전 관리활동에 영향을 미치는 요인을 파악하여 간호사의 환자안전 관리활동 중재 프로그램 개발을 위한 기초자료를 제공하고자 시도되었다. 본 연구결과 환자안전 문화인식과 간호순회인식은 간호사의 환자안전 관리활동에 유의한 영향을 미치는 것으로 나타났다. 따라서 상급종합병원 병동 간호사의 환자안전 관리활동의 수준을 높이기 위해 간호사에 대한 적정한 인력 수준의 배치와 업무량 배분, 업무성과에 따른 적절한 대우와 보상 제공으로 직무 스트레스를 감소시키고 환자안전에 대한 교육을 반복적으로 제공하여 긍정적인 환자안전 문화인식을 확립하도록 의료기관과 국가차원에서 지원하는 것이 필요하다. 또한 간호순회를 통한 간호사 측면의 이익을 보다 잘 인식할 수 있도록 교육 프로그램 등의 중재를 통해 간호순회인식의 수준을 높여 환자안전 관리활동을 향상시키는 것이 필요하다.

이상의 결과를 바탕으로 다음과 같이 제언하고자 한다. 본 연구는 일부 지역의 상급종합병원 병동 간호사만을 대상으로 하였으므로 연구결과의 해석에 신중을 기할 필요가 있으며, 추후 병원 및 간호사 수를 확대하여 간호사의 환자안전 관리활동 실태를 파악하는 후속연구를 제언한다. 본 연구 결과 잡 크래프팅은 상급종합병원 병동 간호사의 환자안전 관리활동에 영향을 미치지 않았으므로 추후 객관적으로 파악할 수 있는 도구로 측정하여 잡 크래프팅이 상급종합병원 병동 간호사의 환자안전 관리활동에 영향을 미치는 요인으로서 작용하는지 반복 연구해 볼 필요가 있다. 또한 잡 크래프팅은 환자안전 문화인식과 간호순회인식에 강한 양의 상관관계를 나타내어 추후 잡 크래프팅이 매개변수로서 환자안전 관리활동에 영향을 미치는지 파악하는 연구를 제언한다. 앞서 살펴보았듯이 간호사의 인력 수준, 환자안전에 대한 교육 정도가 환자안전 관리활동에 많은 영향을 미칠 것이라 생각되므로 일반적 특성으로 간호사 인력 수준, 환자안전 교육정도를 포함하여 환자안전 관리활동의 영향요인을 파악하는 연구를 제언한다. 본 연구결과를 바탕으로 상급종합병원 병동 간호사의 환자안전 관리활동 향상시키기 위한 프로그램을 개발 및 적용하여 그 효과를 검증하는 후속 연구를 제언한다.

## Figures and Tables

**Table 1.** General Characteristics and Patient Safety Management Activities (N = 159)

Variables	Categories	n (%)	M ± SD	Patient safety management activities	
				M ± SD	t/F (*p*)
Gender	Men	4 (2.5)		4.63 ± 0.32	1.80 (.073)
	Women	155 (97.5)		4.25 ± 0.41	
Age (yr)	<30	104 (65.4)	23.35 ± 5.83	4.28 ± 0.44	0.82 (.412)
	≥30	55 (34.6)		4.22 ± 0.36	
Marital status	Single	124 (78.0)		4.25 ± 0.42	-.130 (.897)
	Married	35 (22.0)		4.26 ± 0.39	
Education level	College	12 (7.5)		4.18 ± 0.41	0.90 (.406)
	University	138 (86.8)		4.25 ± 0.42	
	Master	9 (5.7)		4.42 ± 0.28	
Clinical experience (mon)	< 83	103 (64.8)	82.41 ± 73.36	4.28 ± 0.43	1.11 (.268)
	≥ 83	56 (35.2)		4.21 ± 0.37	
Experience in current unit (mon)	< 45	87 (54.7)	44.29 ± 38.06	4.27 ± 0.42	0.51 (.608)
	≥ 45	72 (45.3)		4.24 ± 0.41	
Work unit	Surgery ward	63 (39.6)		4.29 ± 0.41	0.43 (.647)
	Medical ward	84 (52.8)		4.23 ± 0.42	
	Integrated nursing care ward	12 (7.6)		4.30 ± 0.37	

M = Mean; SD = Standard deviation.

**Table 2.** Degree of Perceptions of Patient Safety Culture, Job Crafting, Perceptions of Patient Rounding, and Patient Safety Management Activities (N = 159)

Variables	M ± SD	Actual range	Reference range
Perceptions of patient safety culture	3.88 ± 0.43	2.49~4.97	1.00 ~ 5.00
Leadership	4.09 ± 0.50	2.00~5.00	
Teamwork	4.04 ± 0.51	2.00~5.00	
Patient safety knowledge and attitudes	4.25 ± 0.47	1.80~5.00	
Patient safety policy and procedures	3.91 ± 0.60	2.25~5.00	
Non-punitive environment	3.39 ± 0.78	1.25~5.00	
Patient safety improvement system	3.70 ± 0.52	2.25~5.00	
Patient safety priority	3.15 ± 0.77	1.33~5.00	
Job crafting	3.93 ± 0.64	2.33~5.80	1.00 ~ 6.00
Task crafting	3.90 ± 0.78	1.00~6.00	
Cognitive crafting	4.04 ± 0.85	2.00~6.00	
Relational crafting	3.93 ± 0.77	2.20~6.00	
Perceptions of patient rounding	3.60 ± 0.30	2.93~4.50	1.00 ~ 5.00
Communication	3.57 ± 0.47	2.38~4.44	
Nurse benefit	3.50 ±0.34	2.56~4.44	
Patient benefit	3.76 ±0.33	3.00~4.80	
Patient safety management activities	4.25 ±0.41	3.00~5.00	1.00 ~ 5.00
Patient identification	4.25 ±0.43	3.00~5.00	
Verbal prescription	4.28 ±0.63	2.33~5.00	
Medication nursing	3.93 ±0.50	2.71~5.00	
Operations/Procedures	4.30 ±0.57	3.00~5.00	
Safety environment	4.27 ±0.59	2.67~5.00	
Infection prevention	4.49 ±0.51	3.00~5.00	
Fall prevention	4.59 ±0.52	3.00~5.00	
Pressure ulcer prevention	4.58 ±0.54	3.00~5.00	
Emergency preparedness	4.22 ±0.58	2.71~5.00	

M = Mean; SD = Standard deviation.

**Table 3.** Correlations of Perceptions of Patient Safety Culture, Job Crafting, Perceptions of Patient Rounding, and Patient Safety Management Activities (N = 159)

Variables	Perceptions of patient safety culture	Job crafting	Perceptions of patient rounding	Patient safety management activities
	r (*p*)			
Job crafting	.38 (< .001)	1		
Perceptions of patient rounding	.55 (< .001)	.31 (< .001)	1	
Patient safety management activities	.60 (< .001)	.16 (.043)	.54 (< .001)	1

**Table 4.** Influence of Perceptions of Patient Safety Culture, Job Crafting, and Perceptions of Patient Rounding on Patient Safety Management Activities (N = 159)

Variables	B	SE	β	t	*p*	Tolerance	VIF
(Constant)	1.21	.31		3.87	< .001		
Perceptions of patient safety culture	0.45	.07	.47	6.28	< .001	.64	1.54
Job crafting	-0.07	.04	-.11	-1.78	.076	.83	1.19
Perceptions of patient rounding	0.43	.10	.31	4.31	< .001	.68	1.46
R^2^ = .44, Adjusted R^2^ =.43 (F = 39.71, *p* < .001)							

SE = Standard error; VIF = Variance inflation factor.

## Data Availability

Please contact the corresponding author for data availability.
